# A Data-Informed Perspective on Public Preferences for Retaining or Abolishing Biannual Clock Changes

**DOI:** 10.1177/07487304221096390

**Published:** 2022-05-20

**Authors:** Andrew N. Coogan, Shannon Richardson, Sudha Raman

**Affiliations:** Department of Psychology, Maynooth University, Maynooth, Ireland

**Keywords:** daylight saving time, standard time, chronotype, social jetlag

## Abstract

Scientific, public, and political discourse around the perennial changing of the clocks during the transitions into and out of daylight saving time (DST) is a touchstone issue for the translation of fundamental chronobiology into societal impacts. The Society for Research on Biological Rhythms, along with other sleep science bodies, has issued a position statement that advocates for the abolition of the biannual clock changes and the adoption of permanent standard time for the optimization of population circadian health. However, there is a paucity of data on preexisting public perceptions and preferences with regard to these issues. In this perspective, we examine 5 issues that we believe are pertinent for chronobiologists to consider to enable effective advocacy on these policies; in particular, we discuss public preference for permanent DST and steps that may need to be taken to understand this preference. We inform our discussion with reference to cross-sectional studies we undertook in Spring 2020 and Fall 2019, around the transition out of and into DST Ireland. We conclude that there appears to be a gap between existing public perceptions and preferences around the clock changes and chronobiological and sleep science-informed positions, and that the chronobiology community may benefit from interdisciplinary collaboration with colleagues with specific social sciences expertise to most effectively advocate for these research-informed positions.

The perennial clock adjustments into and out of daylight saving time (DST)/summertime has been a feature of many jurisdictions for over 100 years. Recently, the question of whether such transitions are appropriate has been the focus of considerable scientific, societal, and political discourse in many countries; the European Commission (2018) launched a survey in 2018 on preferences for abolition of the transition to DST, which was followed by a vote in the European Parliament in favor of abolition of the perennial clock change. Similar discussions are contemporary in a number of states of the United States (e.g., the “Sunshine Protection Act” proposed to the US senate in 2019), and in Canada, the Province of Alberta recently held a referendum on a proposition to abolish the clock change and adopt permanent DST, which was very narrowly defeated (49.8% in favor vs 50.2% against; Elections Alberta, 2021).

Arguments in favor of DST emphasize extension of the working day, energy savings from reduced demand for evening electric lighting, recreational amenity resulting from perceived longer hours of evening sunshine, increased opportunities for commerce, and perceived road traffic safety improvements (Committee on Science, 2001). Concerns about DST center on increased desynchrony between internal circadian time and societal schedules (social jetlag; Roenneberg et al., 2019b) as well as sleep, circadian, and health disruptions during the transition to/from DST (Toth Quintilham et al., 2014; Lahti et al., 2008; Tonetti et al., 2013; Fritz et al., 2020). These concerns have prompted professional bodies such as the Society for Research on Biological Rhythms (Roenneberg et al., 2019a) and the American Academy of Sleep Medicine (Rishi et al., 2020) to issue position statements endorsing the abolition of DST and the adoption of year-round standard time.

Given the importance of these questions for the translation of fundamental chronobiology into societal-level policy, the paucity of data on public perceptions of clock changes is striking. From the limited available data, a potential divergence between scientific consensus and public opinion has been noted (Blume and Schabus, 2020). Public perceptions may be of particular importance in debates around clock changes, as changes would be enacted by politicians who may place more weight on public preferences than on the scientific advice.

In the current perspective, we aim to unpack some of the issues that may enable chronobiologists to better address public perceptions around clock changes. To inform our perspective, we reference results from a cross-sectional study of 797 adults resident in Ireland collected after the transition to DST in Spring 2020 and a pilot study of 172 Irish residents collected around the transition to standard time in 2019 (details of the methods and results of these studies can be found in the Supplementary Materials).

## Most People Prefer to Abolish Clock Changes and Adopt Permanent DST

In Spring 2020, 55% reported positive experience of the annual switch to DST, 58% expressed preference to abolish the clock change, and for those respondents ~59% expressed preference for permanent DST (Suppl. Fig. 1). Results from Fall 2019 were very similar (57% positive experience of the switch to DST, 61% preferred abolition of the clock change, and 52% preferred adoption of year-round DST; Suppl. Fig. 2). Participants with negative experience of the annual switch to DST or standard time were markedly more likely to express preference for clock change abolition, and participants with positive experience of the switch to DST, or negative appraisal of the switch to standard time, were more likely to express a preference for adoption of permanent DST (Suppl. Figs. 3 and 5).

The preference for abolition of clock changes is in line with polling by the Irish Government (66% in favor) and the European Commission (84%), as is the preference for permanent DST (77%; Irish Department of Justice and Equality, 2019). A recent study of ~47,000 Norwegian respondents reported that 78.2% were in favor of abolishing the clock change and 61% were in favor of adopting permanent DST (Bjorvatn et al., 2021). As such, it appears that there are clear public preferences to abolish the clock change and adopt permanent DST in the data currently available. It does not seem from our data that the timing of the survey is a major factor in the responses: results from the Spring 2020 and Fall 2019 surveys are very similar in terms of the results for preference to abolish the clock change and adoption of permanent DST (61% in favor of abolition of clock changes and 52% preferring year-round DST). Whether such preferences are stable within individuals across time can only be assessed with prospective longitudinal studies.

The issue of permanent DST as the “landing zone” is potentially strongly confounded by a number of factors. First, positive appraisal of DST may be based on positive appraisal of summer, rather than DST per se. This is a difficult issue to address as the current participants will not have experienced DST outside of the context of late Spring/Summer/early Fall, or standard time during the summer. It is not obvious how DST can be de-confounded from summer; one possibility is that rather than presenting the question as “DST or Standard Time,” the options are framed in terms of prospective dawn and dusk clock times under the possible arrangements. However, responses to such framing could be biased by the selection of the reference periods to be presented (e.g., during high summer vs mid-fall/spring). There is also a potential role of geography, as these concerns may be lessened for territories with less seasonal variation in natural photoperiod. Second, the mismatch in comparing an arrangement of which a respondent has direct experience of with a prospective arrangement of which a respondent has no direct experience of may lead to positivity bias through errors of hedonic forecasting (Kahneman and Thaler, 2006). Furthermore, increased social jetlag and its detrimental impacts on health may be an “invisible” problem (i.e., not immediately apparent to those experiencing it and consequences of which are delayed in time and maybe subject to temporal discounting; Story et al., 2014), while other aspects such as lesser amounts of evening sunlight according to clock time are proximal and directly observable events; as such these factors may be hedonically evaluated differentially. As such, chronobiologists may benefit from collaboration with colleagues in social sciences that may help frame contingencies around prospective arrangements were the biannual clock changes to be abolished.

## Being in DST Versus Switching to DST

The framing of the questions used in our survey refers to experience of “switching” between standard time and DST, while the questions on what arrangement comes into force were the switches to be abolished refers to being under permanent DST or standard time (by definition, the switching would no longer occur). This highlights an important consideration: the distinction between transient effects of the switches versus the subsequent ongoing arrangement. In chronobiological terms, this may be framed as re-entrainment in the immediate weeks following the clock change versus ongoing increased circadian desynchrony/social jetlag under DST. This distinction may be important in understanding the adverse health and behavioral consequences associated with the spring transition to DST, and may be a contributor to the recent suggestion that the cardiovascular risk associated with the spring change to DST is understated due to the selection of inappropriate reference periods during which circadian desynchrony will still be high (Čulić and Kantermann, 2021).

While this may be an important chronobiological distinction, it is unlikely to be one that a majority of the public will make. In our survey, it is unclear whether negative experiences of the switch to standard time actually refers to (a) the transient inconvenience of the switch period itself, (b) the ongoing experience of being in standard time/DST, or (c) the confounded association of standard time with winter or DST with summer (or any combinations of (a), (b), and (c)). Furthermore, being in standard time or DST is not a uniform experience given seasonal changes in natural photoperiod: for example, in Ireland under standard time, the hours of daylight per 24 h vary between 7.5 h in December and 12.5 h in April. As such, chronobiologists should be more explicit in their aims when engaging with the public or policy makers; is it to abolish the transient effects of the clock changes or to decrease social jetlag through the adoption of permanent standard time, or is it both? This is a highly pertinent issue given proposals in various jurisdictions where the options presented to the public are narrowed to yes/no questions on abolishing the clock changes and adopting permanent DST, potentially resulting in less chronobiologically favorable scenarios due to detrimental effects of increased year-round social jetlag (e.g., poorer sleep health, impaired mood and cognition, poorer cardiometabolic health) outweighing the benefits of eliminating the transitions (decreased risk of cardiovascular events and road traffic accidents in the week(s) following clock change). Such a risk/benefit calculation is complicated by uncertainties around the magnitude of effects of social jetlag in relation to health outcomes, and the ongoing discussion on risks associated with the transitions (Čulić and Kantermann, 2021).

## The Place of Geography

A jurisdiction’s longitude within a time zone will influence social jetlag (with more westerly locations associated with greater social jetlag; Roenneberg et al., 2019b) and more adverse health outcomes, lower prosocial behaviors, and lower economic activity (Gu et al., 2017; Holbein et al., 2019; Giuntella and Mazzonna, 2019). Increasing distance from the equator associates with greater seasonal variations in photoperiod; Martín-Olalla (2019, 2022) suggests differential need for DST transitions in jurisdictions above 50° circle of latitude compared with those under 47°, and as such public experience of DST may vary depending on the magnitude of the seasonal change in natural photoperiod, with greater seasonal changes in photoperiod possibly associating with adverse effects of permanent DST due to late winter dawn times. For example, under DST, the time of sunset in Dublin at the summer solstice is approximately 10 p.m. and there is ~17 h of daylight, while the standard time sunset at the winter solstice is 4:15 p.m. after only 7.5 h of daylight. In comparison, those same local values for another European capital city, Rome, are sunset at 8.50 p.m. after ~15.25 h of sunlight in the high summer and sunset at 4:45 p.m. with ~9.1 h of daylight in late December. The loss of 1 h of daylight in high summer in year-round standard time may be more negatively appraised in Rome than it is in Dublin, while Dubliners will experience short hours of winter daylight irrespective of how social activity is synchronized to sun time.

In our data sets, we did not find any significant associations between either longitude or latitude with any clock change items; however, Ireland is a small country. Bjorvatn et al. (2021) reported an association between northerly and easterly location within Norway with stronger preference for abolition of the clock changes. The results of the recent referendum in Alberta, which are available at an electoral district/municipal level (Elections Alberta, 2021), may afford a further analysis of the influences of latitude and longitude on preferences expressed in a “real-world” exercise (although such preferences will be constrained by the nature of the question asked: biannual clock changes vs permanent DST). See Antle et al., 2022.

Finally, due consideration should be given to geopolitical considerations that apply to any territory. For example, given that Northern Ireland is part of the United Kingdom which has stated that it will not be altering its clock change regime, the risk of the Republic of Ireland being in a different time zone to Northern Ireland may shape preferences, with a survey by the Irish Government indicating that 82% of respondents would not favor a solution that imposed different time zones in the Republic of Ireland and Northern Ireland. Such regional political considerations are likely to be of importance in shaping public attitudes in other international and federal jurisdictions. Finally, there have been no studies examining factors such as conspiratorial thinking and trust in government and science (Pennycook et al., 2020) on attitudes to clock changes. It appears to the authors that in some jurisdictions, the clock change debate has become politically polarized, and as such collaboration with social scientists with expertise in researching and shaping public discourse in such political contexts may benefit the chronobiology community in advocating for arrangements that are optimal for circadian and sleep health.

## Chronotype Does Not Matter Much

Another important question is which factors inform individual opinions. In our Spring 2020 study, the most frequently endorsed reason informing preference to keep or abolish the clock changes was “health” for those favoring abolition, while those favoring retention of the clock changes most frequently endorsed “leisure activity in the evening” (Suppl. Fig. 6). This may indicate that chronobiologists and sleep scientists have been successful in communicating health concerns around clock changes, and that these concerns have entered into public consciousness (e.g., Roenneberg et al., 2019a). Perhaps a concerted effort is now required to highlight the potential harms that would be associated with the adoption of permanent DST, given the strong chronobiology-based arguments against such an arrangement (Sládek et al., 2020; Borisenkov et al., 2017).

Another individual-level factor that may influence preferences for DST is chronotype, and levels of social jetlag associated with chronotype. We hypothesized that individuals with later chronotypes would favor solutions that provide more hours of evening daylight, and those with higher levels of social jetlag would favor solutions that would potentially reduce it. In both of our data sets, chronotype and social jetlag only weakly associated with preference for abolition of clock changes (earlier midsleep on free days (MSFsc) and lower social jetlag were associated with preference for abolition; Suppl. Figs. 9 and 10), but there was no association between preference for permanent DST and either MSFsc or social jetlag (Suppl. Figs. 11 and 12). Bjorvatn et al. (2021) reported that evening orientation was associated with a preference for abolition of the clock change, although the majority of all chronotype groupings assessed favored abolition and, with the exception of extreme morning types, endorsed a preference for permanent DST. As such, there is currently no convincing indication that preference for clock changes/permanent DST is influenced by chronotype to any meaningful extent, although individuals with later chronotypes/greater social jetlag may be most adversely impacted by permanent DST (Čulić and Kantermann, 2021). Other factors that may influence preferences for clock changes are gender and age: in our survey, participants favoring abolition of clock changes were somewhat older and slightly more likely to be male, as they were in the Bjorvatn et al. (2021) study. It is not clear why such associations may be present, although both sex and age are important influences on circadian function and chronotype (e.g., Fischer et al., 2017).

## COVID-19 and Clock Changes

At the time of writing, we are over 24 months into the COVID-19 pandemic. There are a number of studies showing that the societal restrictions imposed to mitigate the effects of COVID-19 had profound impacts on sleep timing and have revealed the social pressures under which sleep operates in “normal” circumstances (Korman et al., 2020). For example, in Ireland, the average amount of social jetlag decreased from ~1 h before the pandemic to 36 min in the early phase of the pandemic, with this change associated with later sleep timing on work days and markedly decreased use of alarm clocks (Raman and Coogan, 2022). As we move past the pandemic, changes in work practices such as increased working from home and decreased commuting time may result in decreases in social jetlag. This highlights an important consideration for clock changes: “clock time” is only one mechanism through which societies organize their behaviors relative to sun time, with other factors being local work, school and university start times, and the organization of work-free time. Therefore, increased social jetlag resulting from permanent DST could be offset by counterbalancing changes in school and work start times, decreased commuting times, and increased worker flexibility. Of course, it would be foolish to assume that such steps would automatically follow any change to the clock change arrangements. Furthermore, a recent transnational study indicated that while 46% of respondents showed a decrease in social jetlag during COVID-19, 20% experienced an increase and 44% reported no change (Brandão et al., 2021), and Raman and Coogan (2022) report that the reduction in social jetlag is dependent on requirement to continue to physically attend the place of work. Nonetheless, there could be long-lasting changes to work and education practices post-pandemic that could partially mitigate the effects of DST, although as clock changes impacts on large sections of a population who do not have agency to match their activity to their chronotype (such as school students), societal arrangements would still have primacy. Therefore, we should appropriately address arguments that chronobiologically maladaptive effects of permanent DST would be offset by non-statutory elective changes in working time and societal arrangements.

Another important COVID-19 consideration is that our Spring 2020 survey was conducted in the early phase of the pandemic, and so results may not generalize. However, the results from Spring 2020 were very similar to our Fall 2019 results and also echoed results from other sources (e.g., Bjorvatn et al., 2021); future post-pandemic studies will shed further light on this issue.

## Summary and Conclusion

This perspective did not seek to revisit chronobiological arguments for abolishing clock change and adopting permanent standard time, which is already well articulated (Roenneberg et al., 2019a, 2019b; Rishi et al., 2020), nor was the purpose to discuss the nature of “real world” entrainment to solar time versus social time (Zerbini et al., 2021a, 2021b; Skeldon and Dijk, 2021). Rather, we aimed to examine public perceptions of clock changes, motivated by a paucity of data on the subject. Surely if the chronobiology community is to most effectively engage in the public debate on clock changes, then it must do so in a sufficiently data-informed manner as to what the starting point of such a debate may be? The recent referendum result in Alberta reflects how evenly divided the public may be on the issue of whether to abolish or keep the clock changes and what arrangement to replace it with, and may also offer a salutatory warning of the cost of not preemptively engaging with public perceptions and preferences. It may be uncomfortable for the chronobiology community to consider that an attempt to improve population circadian health by advocating for the abolition of clock changes could result in a more harmful situation of permanent DST. To counter the permanent DST contingency, surely we need to “meet” the public at where they are at, so to effectively translate fundamental circadian science into public policy Blume and Schabus (2020) have noted the perils of attempting to “explain away” public preferences. However, given the paucity of data on what those preferences even are, and what factors shape them, there is a need for further work in various jurisdictions. Furthermore, interdisciplinary collaboration with colleagues with expertise in social sciences could promote effective dissemination of chronobiological science to better influence public and political discourse toward solutions that are optimal for population health and well-being.

## Supplemental Material

sj-docx-1-jbr-10.1177_07487304221096390 – Supplemental material for A Data-Informed Perspective on Public Preferences for Retaining or Abolishing Biannual Clock Changes Click here for additional data file. Supplemental material, sj-docx-1-jbr-10.1177_07487304221096390 for A Data-Informed Perspective on Public Preferences for Retaining or Abolishing Biannual Clock Changes by Andrew N. Coogan, Shannon Richardson and Sudha Raman in Journal of Biological Rhythms
