# Supplementary material for: A Data-Informed Perspective on Public Preferences for Retaining or Abolishing Biannual Clock Changes
Source: J Biol Rhythms. 2022 May 20;37(4):351–7. doi: 10.1177/07487304221096390 (PMC9326796; doi:10.1177/07487304221096390)
Supplement: sj-docx-1-jbr-10.1177_07487304221096390 – Supplemental material for A Data-Informed Perspective on Public Preferences for Retaining or Abolishing Biannual Clock Changes [file sj-docx-1-jbr-10.1177_07487304221096390.docx]

**Supplementary Materials.**

***Survey Method***

Ireland switches to DST (GMT+1) in the spring on the last Sunday in March, and back to standard time on the last Sunday in October (as a note on nomenclature, summertime/DST is officially termed “Irish Standard Time”, although for the avoidance of confusion this was only referred to as summertime/DST as the usage of “Irish Standard Time” does not appear common in Ireland and is at variance with the terminology used in nearly all other jurisdictions). Data was collected from 11^th^ April 2020 to 4^th^ June 2020, approximately two weeks after the clocks switched to DST; this period overlapped with the early stages of the COVID-19 pandemic and the imposition of societal restrictions in Ireland, which was associated with marked changes in sleep timing in Ireland and elsewhere (Korman et al 2020; Raman and Coogan 2021).

Ethical approval was granted by the Research Ethics Committee of Maynooth University and informed consent was indicated by all participants whose data was included in the analysis. Adults living in Ireland were recruited via the Brainstorm blog of the national broadcaster (Radio Television of Ireland), personal contacts, social media posts, and recruitment via Qualtrics Research Participants service from a nationally representative panel (N=500 recruited from this route). Respondents completed a questionnaire on experiences of and prospective preferences for clock change, which was adapted from the EU commission survey (European Commission 2018), as well as providing demographic information such as age, gender, place of residence, employment status and shift-work status. A version of the questionnaire used is included at the end of these Supplemental Materials. The ultra-short version of the Munich Chronotype Questionnaire (μMCTQ; Ghotbi et al. 2020) was used to assess sleep/wake timings and social jetlag. To control for the impact of COVID-19 mitigation measures on sleep/wake behaviors, participants were asked to score their usual sleep/wake behaviors prior to the pandemic. Participants also completed the 16 item Dysfunctional Beliefs and Attitudes about Sleep questionnaire to gauge their cognitions relating to sleep (DBAS; Morin et al. 2007). Participants were also requested to rate their sleep quality prior to the pandemic on a single item of zero to ten, with higher scores indicating better sleep.

Additionally, a separate data set from a pilot study was collected from 14th to the 28th November 2019 (the change from DST to standard time occurred on the 27th October). Data was collected from a total of 201 adults via convenience sampling; when screened for exclusion of shift workers, non-Irish residents and incomplete responses, 172 sets of responses were included for analysis. For this study, individuals completed the full MCTQ (Roenneberg et al. 2012), the DBAS, the Pittsburg Sleep Quality Scale (PSQI; Buysse et al. 1989) as well as the same questionnaire for experiences of and attitudes to clock changes that was subsequently used in spring 2020. As this data set was collected prior to the COVID-19 pandemic, there were no mentions of or adjustments for “lockdown” measures.

For statistical analysis, Chi-square tests for independence were used to assess associations between categorical variables, *t*-tests or one-way analysis of variance were used for normally distributed dependent variables or Mann-Whitney U or the Kruskal-Wallis Test for non-normal dependent variables were employed to assess between-groups differences, with *P*<0.05 as indicating a statistically significant effect. Due to differences in methodology and sample sizes between the spring 2020 study and the fall 2019 pilot study, direct inferential comparisons were not undertaken between the cohorts. Effect sizes were expressed as r for non-parametric tests, Cohen’s d for parametric tests and Cramer’s V for chi-square tests. Whilst the majority of the analysis conducted was descriptive or exploratory, there were a number of pre-specified hypothesis that were tested through inferential statistics: that preference for clock change abolition and DST would associated with later chronotype and greater dysfunctional attitudes and beliefs about sleep and social jetlag.

***Survey Results***

After exclusion of shift workers, responses from 797 participants in spring 2020 were included in the analysis (mean age 40.2 years +0.45, 62.4% female; Table S1 for further sample descriptive statistics). Regarding subjective perceptions of the switch from standard time to DST in March, 54% reported positive experience, 17% reported negative experience and 29% had no opinion (Supplementary Figure 1A). Regarding subjective perceptions of the switch from DST to standard time in October, 41% reported negative experience, 31% reported positive experience and 28% had no opinion (Supplementary Figure 1B). 26% of participants reported positive experience of the DST switch, but negative experience of the standard time switch, 5.5% had negative experience of the DST switch but positive experience of the standard time switch, 22% had negative experiences of both switches and 9.9% had negative experiences of both switches. With respect to the potential abolition of the biannual clock change, 59% expressed preference to abolish the clock change (Supplementary Figure 1C). For respondents who indicate a preference for abolition of the clock change, 59% expressed preference for permanent DST, 25% expressed preference for permanent standard time, and 16% expressed no opinion (Supplementary Figure 1D). Results from the fall 2019 data set showed very similar results:, 57% expressed positive experience of the switch to DST, 46% expressed negative experience of the switch to standard time, 61% preferred abolition of the clock change and 52% preferred adoption of year-round DST (Supplementary Figure 2).

Participants’ preference to keep or abolish clock switching had a moderate strength association with their experience of the yearly switch to DST (*X*^2^= 53.2, *P*<0.001, Cramer’s V=0.25); participants with a negative experience of the switch to DST were strongly more likely to favor clock change abolition than clock change retention (Supplementary Figure 3). Similarly, there was a significant strong association between preference for abolishing the clock change and experience of the annual switch to standard time (*X*^2^= 120.3, *P*<0.001, Cramer’s V=0.389), with those with a negative experience of the transition to standard time more likely to express a preference for abolition of the clock change (Figure 2B). For participants expressing a preference to abolish the annual clock change (N=473), preference for permanent DST or standard time was weakly associated with experience of the biannual clock changes; participants with positive experience of the switch to DST were more likely to express a preference for permanent DST and those with an existing negative experience of the switch to standard time were also more likely to express a preference for permanent DST (experience of switch to DST: *X*^2^=41, *P*<0.001, Cramer’s V=0.21, Supplementary Figure 3C; experience of switch to standard time *X*^2^= 37.9, *P*<0.001, v=0.2; Figure Supplementary Figure 3D). For those participants who endorsed a preference for keeping the clock changes (N=324), preference for permanent DST (were there to be abolition of the clock change) was strongly associated with positive experience of the switch to DST (*X*^2^=28, *P*<0.001, Cramer’s V=0.21) whilst preference for permanent DST was also associated with a positive experience of the switch to standard time *X*^2^=26, *P*<0.001, Cramer’s V=0.20; Supplementary Figure ). Similar results were found in the Fall 2019 data set, with preference for abolition of the clock change associating moderately with negative experience of the switch to DST (*X*^2^= 17.9, *P*<0.001, Cramer’s V=0.32) and associating strongly with standard time (*X*^2^= 29.9, *P*<0.001, Cramer’s V=0.42; Supplementary Figure 3 A+B). Further, in the Fall 2019 data set preference for year-round DST was also moderately associated with positive experience of the switch to DST (*X*^2^=20.7, *P*<0.001, v=0.244; Supplementary Figure 3C) and with negative experience of the switch to standard time (*X*^2^=24.1, *P*<0.001, v=0.263; Supplementary Figure 3D).

We examined the association of employment status with both preference for abolition and the adoption of year-round DST or standard time in Spring 2020; those in permanent regular employment showed clear preference for abolition and permanent DST, although comparison with other categories was not meaningful given the small numbers in of respondents in each of those (Supplementary Figure 4). When asked to rank the importance for their preferred option to retain or abolish the biannual clock change, respondents with a preference for abolishing the change rated their preference more highly than those who expressed preference for retaining the clock change, although the magnitude of the difference was small (median of 6 for abolish vs. 5 for retain on a scale of 1-10, p=0.004 by Mann-Whitney U test, r=0.118; Figure 3A). Those who preferred abolition of the clock change were also somewhat older (mean age 41.4 +0.6 years for abolish vs. 38.3 + 0.7 years for keep; P=<0.001 by T-test, Cohen’s d= 0.25; Supplementary Figure) and were slightly more likely to be male (*X*^2^=6.5, *P=*0.038, Cramer’s V=0.09; 190 males in favor of abolition vs 108 for retention, 283 females in favor of abolition vs 214 for retention). For reasons relating to their preference for retaining or abolishing the clock changes, those who preferred retention indicated “Leisure Activities in the Evening” as the most frequently endorsed top reason for the preference (~36%), followed by “Health” (~21%; Figure 3). For those who expressed a preference for abolishing the clock change, the most frequently endorsed top reason for this preference was “Health” (~40%) followed by “Leisure Activities in the Evening” (~28%; Figure 3). These differences in the endorsed leading reasons for the keep/abolish preferences were statistically significant and of moderate size (*X*^2^=54.6, *P<*0.001, Cramer’s V=0.262).

When sleep and chronobiological variables were assessed across attitudes around maintaining or abolishing the biannual clock change, social jetlag was found to vary (with a small effect size), according to abolish/keep preference, with those expressing preference to abolish the clock change experiencing less social jetlag (1h 2min + 0.04 for abolish vs. 1h 14min + 0.05 for retain; *P*=0.003 by Mann-Whitney U test, r=0.11; Supplementary Figure 9). Respondents who preferred abolishing the clock change also had slightly earlier midsleep on work-free day (MSFsc) than those who preferred retention (04:02h + 0:03 vs 04:09h + 0:04, *P*=0.02 by t-test, Cohen’s d=0.17; Supplementary Figure 9). Similar associations were also found in the Fall 2019 data set (social jetlag of 1h 13min + 0.09 for abolish vs. 1h 31min +0.13 for retain, P=0.045 by Mann-Whitney U test; MSFsc of 04:00h + 0:07 for abolish vs. 04:17h + 0:12 for retain, P=0.036 by t-test; Supplementary Figure 7). There were no statistically significant differences between those that preferred abolition of the clock change versus retention on average weekly sleep duration (*P*=0.42), subjective sleep quality (*P*=0.40) or dysfunctional attitudes and beliefs about sleep (DABS; *P*=0.89; Supplementary Figures 6 & 7). When data from respondents who preferred abolition of the clock change was examined according to their favored option of what should replace the current regime, there was no effect of preference for permanent DST, standard time, or no opinion responses on social jetlag (*P*=0.97), MSFsc (*P*=0.81), subjective sleep quality (*P*=0.73) or DBAS scores (*P*=0.45; Supplementary Figure 8). Average weekly sleep duration was slightly shorter in those who expressed no opinion versus both those expressing preferences for permanent DST or standard time (7h 32min +0.15 vs.7h 58min +0.08 vs. 8h 2mins +0.19 respectively; *P*=0.011 via Welch test for unequal group sizes, *P*=0.02 between permanent DST and no opinion and *P*=0.013 between permanent standard time and no opinion via Tukey HSD test; all small effect sizes; Supplementary Figure 8). In the Fall 2019 pilot study, there were no statistically significant associations observed between social jetlag, MSFsc, sleep duration, sleep quality or DBAS scores (Supplementary Figure 9).

**Table S1: Respondent Demographics of the Spring 2020 Study.**

| N=894; Mean age = 39.7 (SD=12.7) | |
| --- | --- |
| Gender |  |
| Female | 534 (59.7%) |
| Male | 358 (40.0%) |
| Non-Binary | 2 (0.3%) |
| Age Groups |  |
| 18-25 | 132 (15%) |
| 26-35 | 220 (24%) |
| 36-45 | 248 (28%) |
| 46-55 | 187 (21%) |
| 56-65 | 83 (9%) |
| >65 | 24 (3%) |
| Shift worker | 97 (11%) |

**Table S2: Respondent Demographics of the Fall 2019 Pilot Study.**

| N=172; Mean age = 35.8 (SD=15.1) | |
| --- | --- |
| Gender |  |
| Female | 118 (68.6%) |
| Male | 54 (31.4%) |
| Age Groups |  |
| 18-25 | 76 (44%) |
| 26-35 | 22 (12.8%) |
| 36-45 | 21 (12.2%) |
| 46-55 | 33 (19.2%) |
| 56-65 | 16 (9.3%) |
| >65 | 4 (2.3%) |
|  |  |


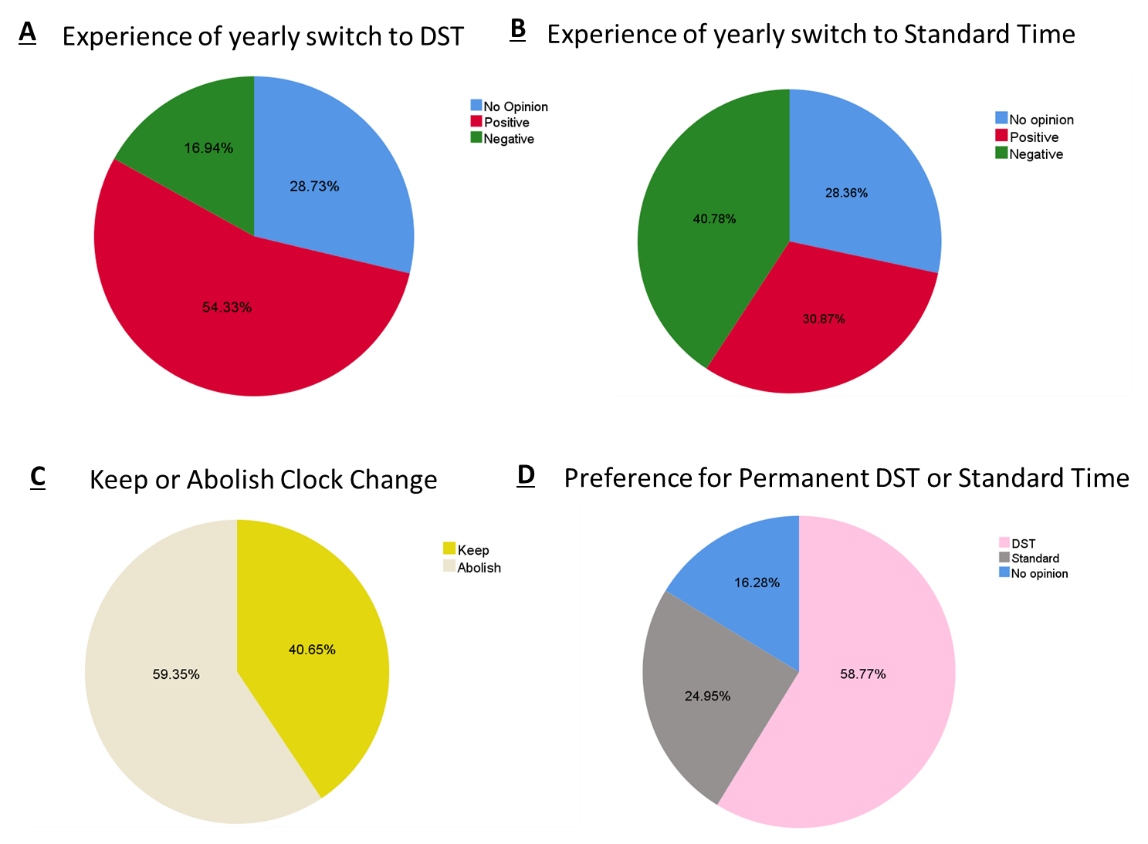


**Supplementary Figure 1**: Pie-charts showing experiences of the yearly change into (A) and out of (B) DST (n=797), attitudes towards keeping or abolishing the yearly clock changes (C) and in those who favored abolition, which were the preferred year-round solution (D; N=473).

#
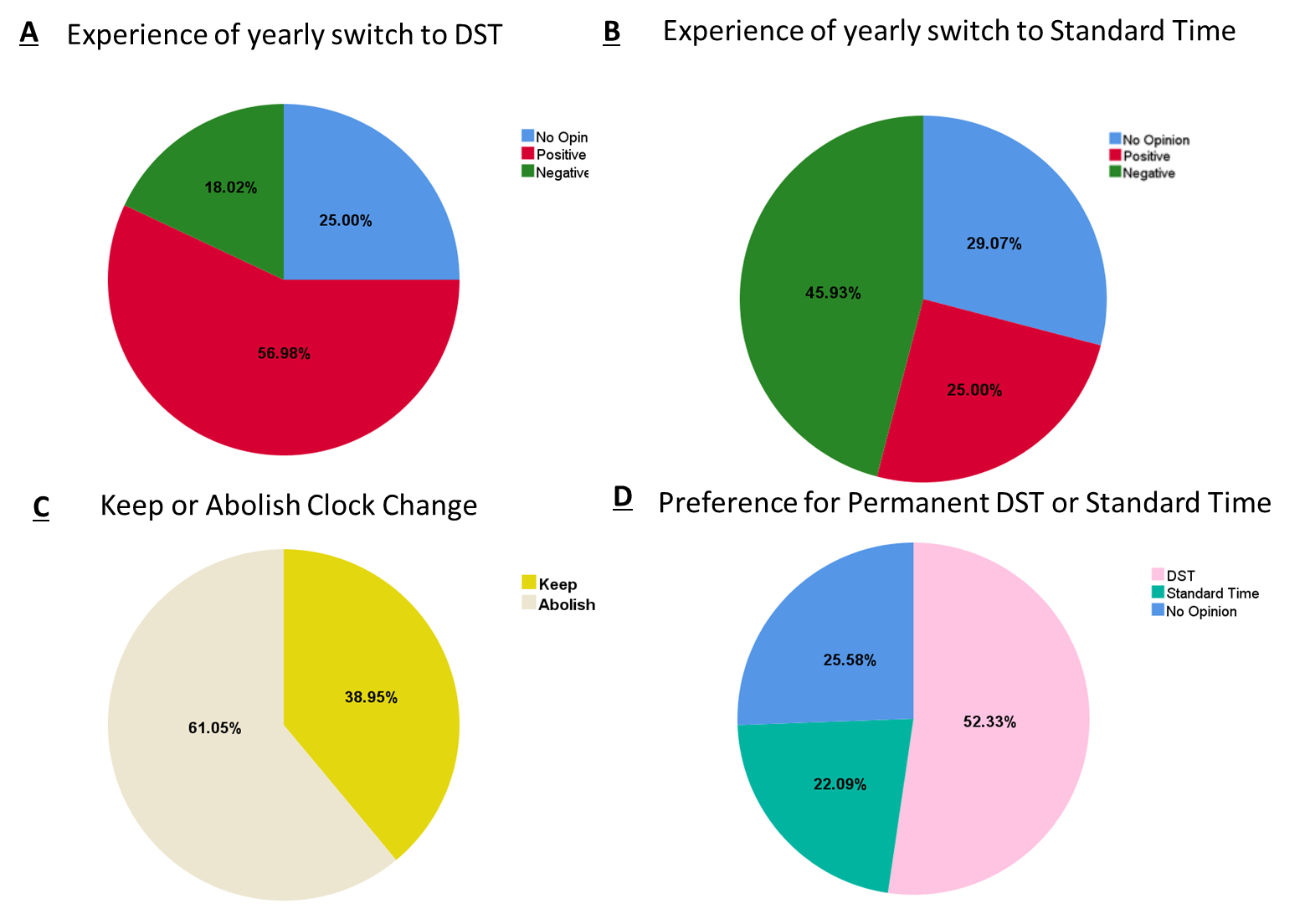


# Supplementary Figure 2: From the Fall 2019 pilot study, pie-charts showing experiences of the yearly change into (A) and out of (B) DST attitudes towards keeping or abolishing the yearly clock changes (C) and which were the preferred year-round solution if clock changes were abolished (D; N=172).


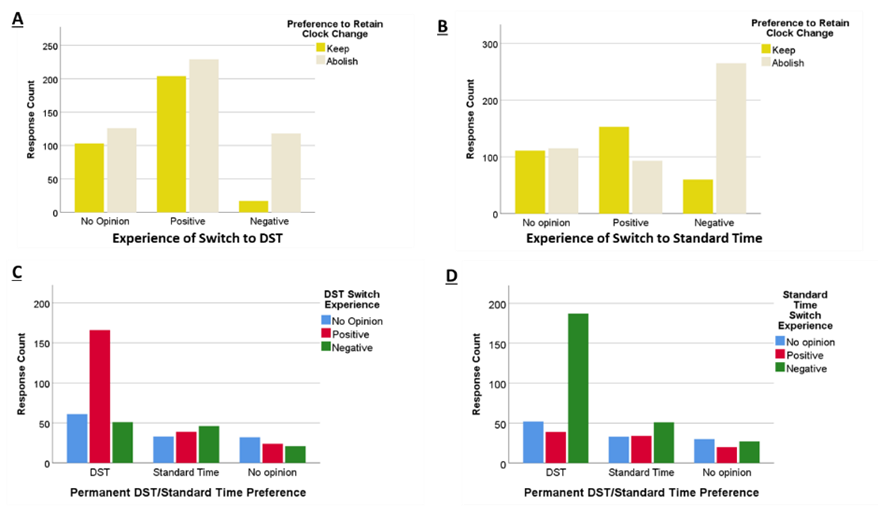


**Supplementary Figure 3**: Bar graphs showing the association between experience of the yearly switch into (A) and out of DST (B) with preference to keep/abolish the perennial changes and for those preferring abolition with the adoption of year-round DST or standard time (C, D).

#
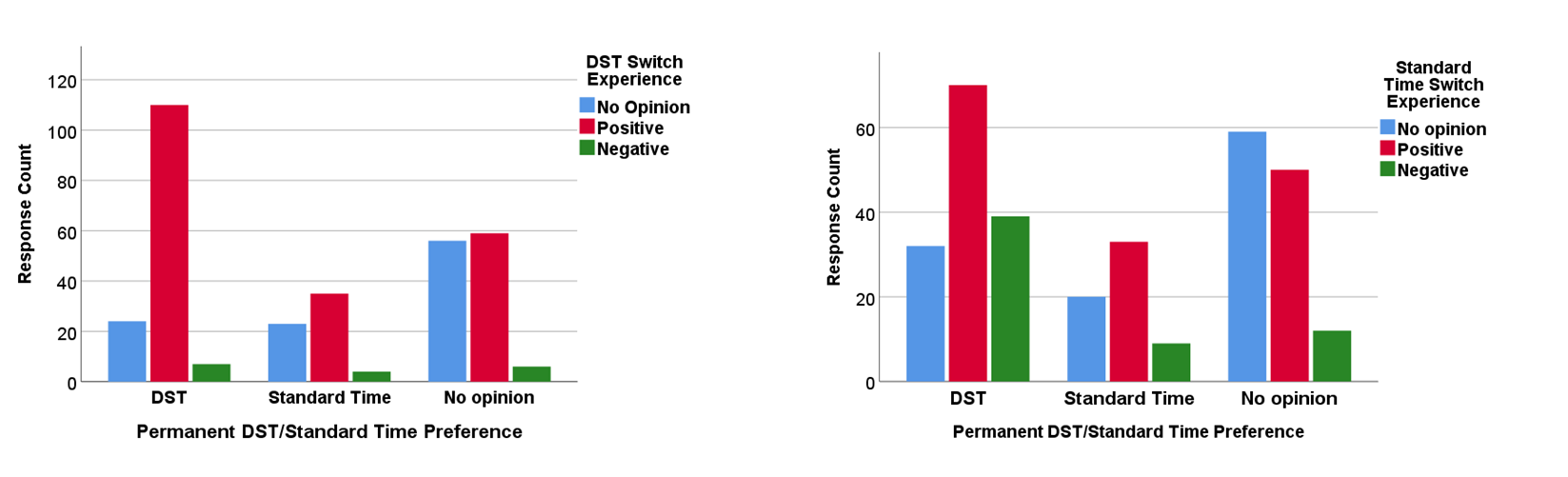


**Supplementary Figure 4**: From the Spring 2020 study, bar graphs showing the association between experience of the yearly switch into and out of DST with preference for the adoption of year-round DST or standard time for participants who expressed preference for retention of the clock changes (N=324).


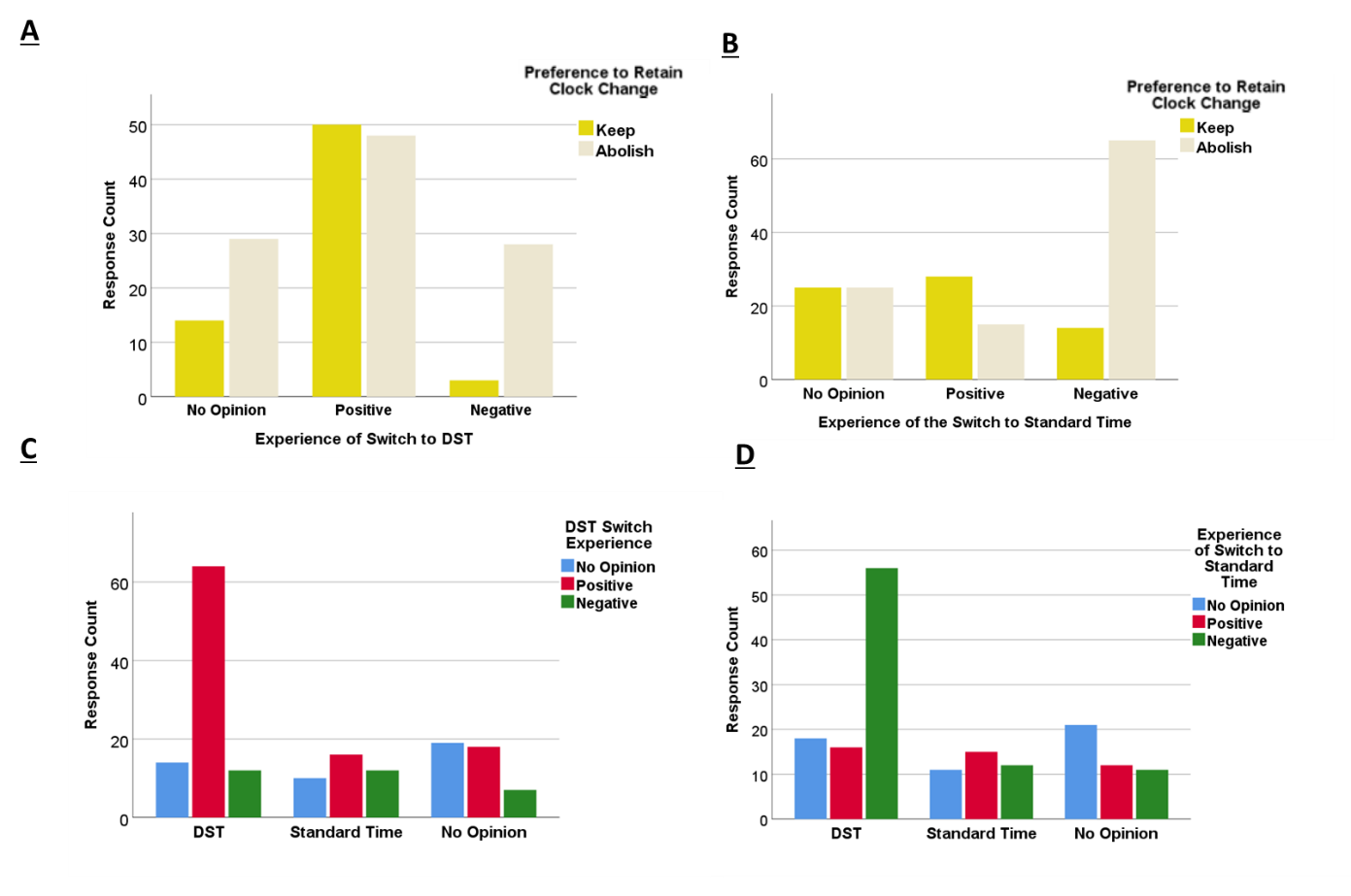


**Supplementary Figure 5**: From the Fall 2019 pilot study, bar graphs showing the association between experience of the yearly switch into (A) and out of DST (B) with preference to keep/abolish the perennial changes and for adoption of year-round DST or standard time (C, D; N=172).


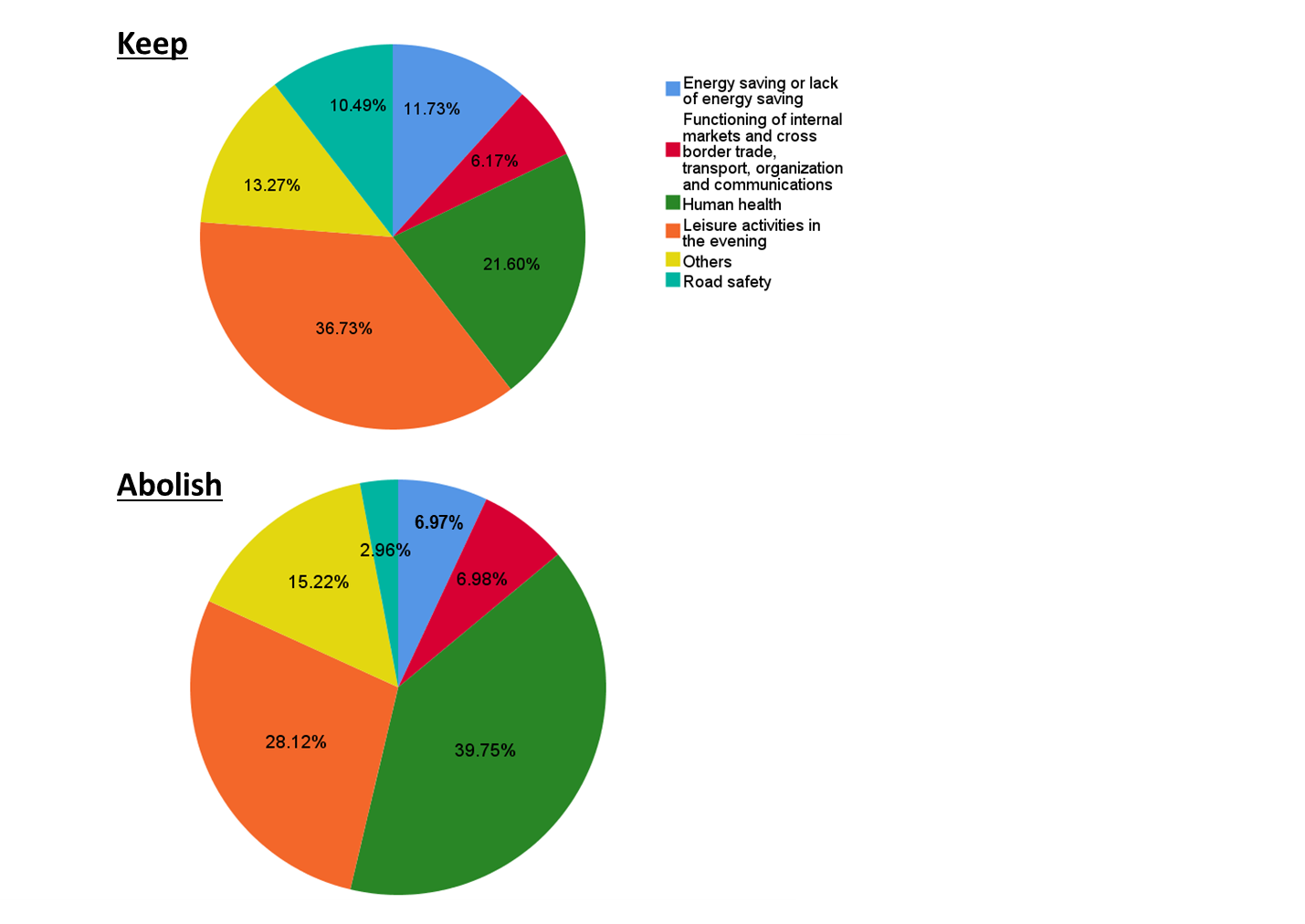


**Supplementary Figure 6**: Pie charts indicating the reasons endorsed as most important for the expressed preference to keep or abolish the perennial clock changes.

**
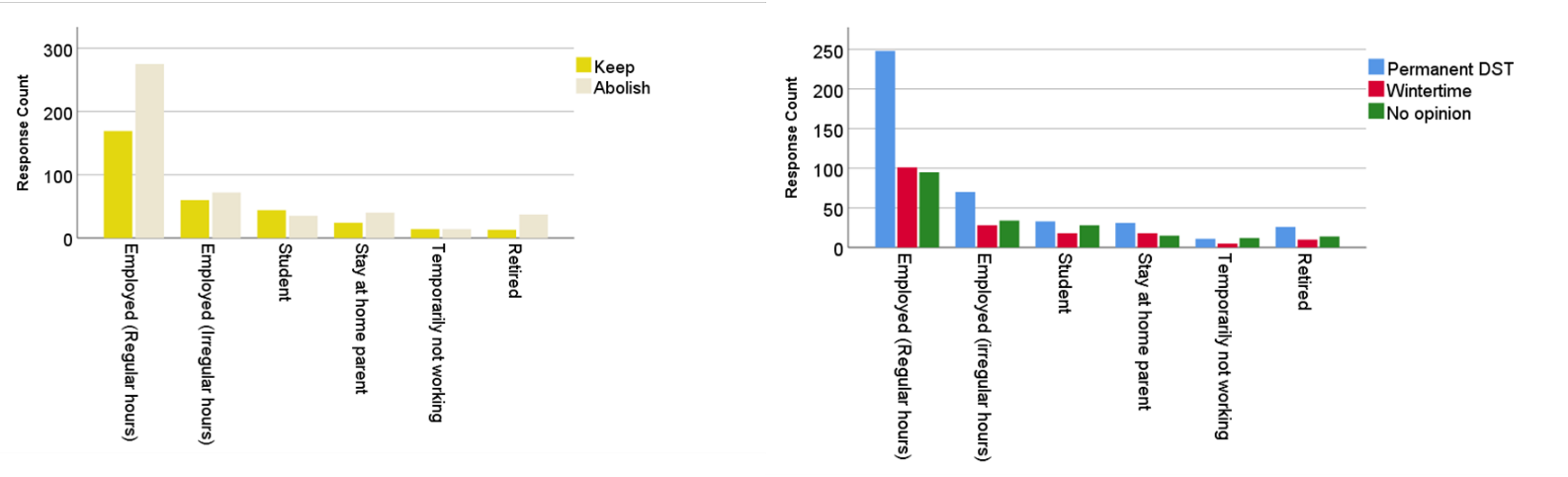
**

**Supplementary Figure 7:** From Spring 2020, the association of employment status with preferences to keep or abolish the clock changes and the adoption of either permanent DST or Standard Time (N=797).


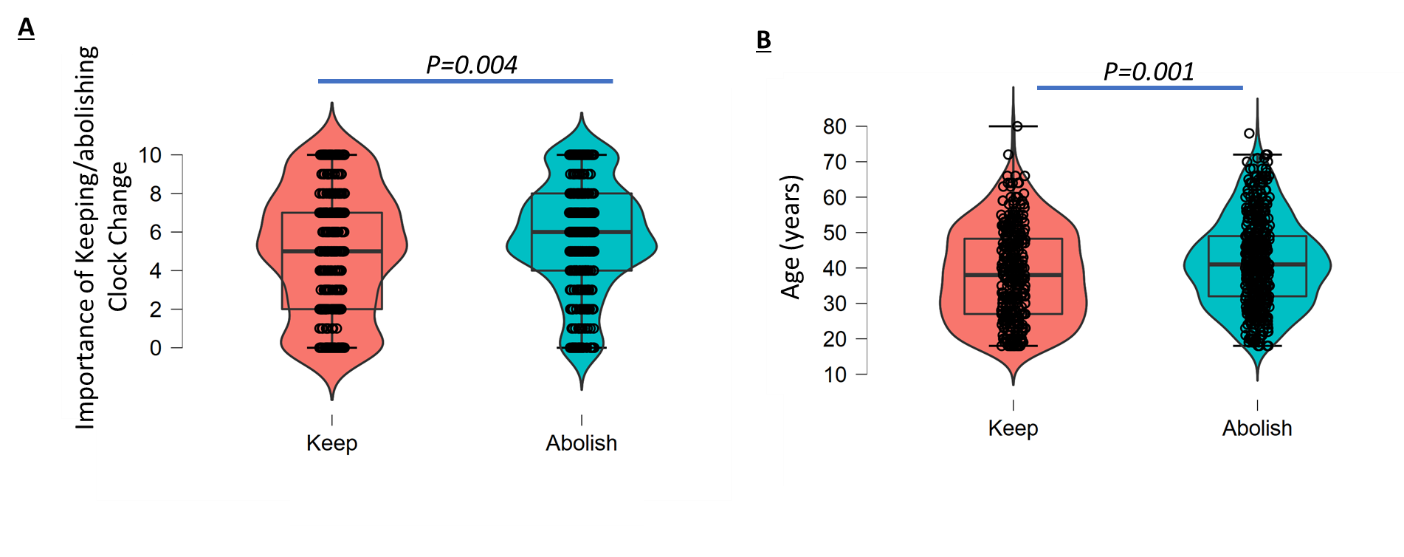


**Supplementary Figure 8**: Box and Violin plots of the ranking of importance of the favored option in those favoring keeping or abolishing the perennial clock changes (A) and the distribution of age across those favoring keeping or abolishing the clock changes (B).


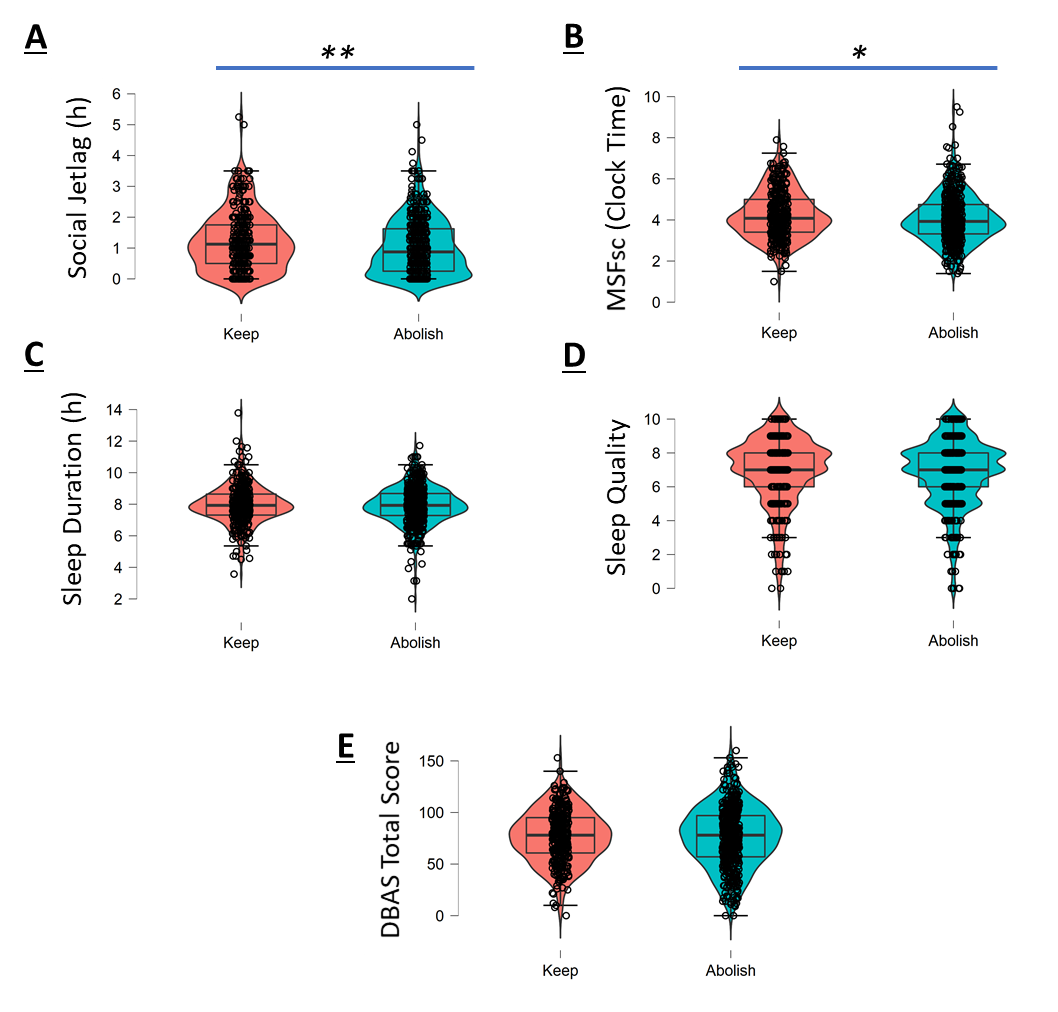


**Supplementary Figure 9:** Box and Violin plots showing the distribution of (A) social jetlag, (B) MSFsc, (C) average weekly sleep duration, (D) subjective sleep quality and (E) DBAS scores in participants wither favoring retention or abolition of the clock changes. ** indicates P<0.01, * indicates P<0.05.


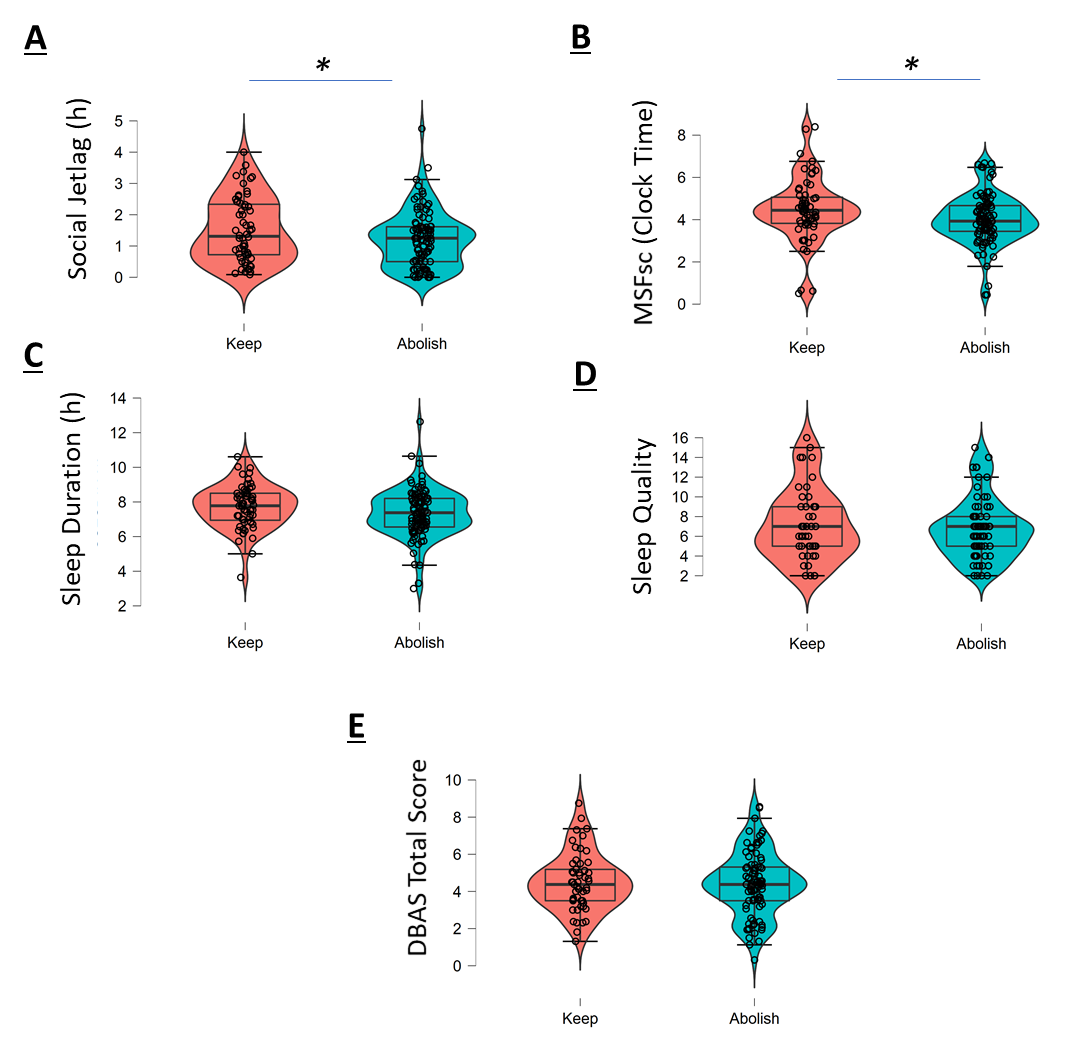


**Supplementary Figure 10:** From the Fall 2019 study, box and violin plots showing the distribution of (A) social jetlag, (B) MSFsc, (C) average weekly sleep duration, (D) subjective sleep quality as measured by overall PSQI score and (E) DBAS scores in participants either favoring retention or abolition of the clock changes. * indicates P<0.05, (N=172).


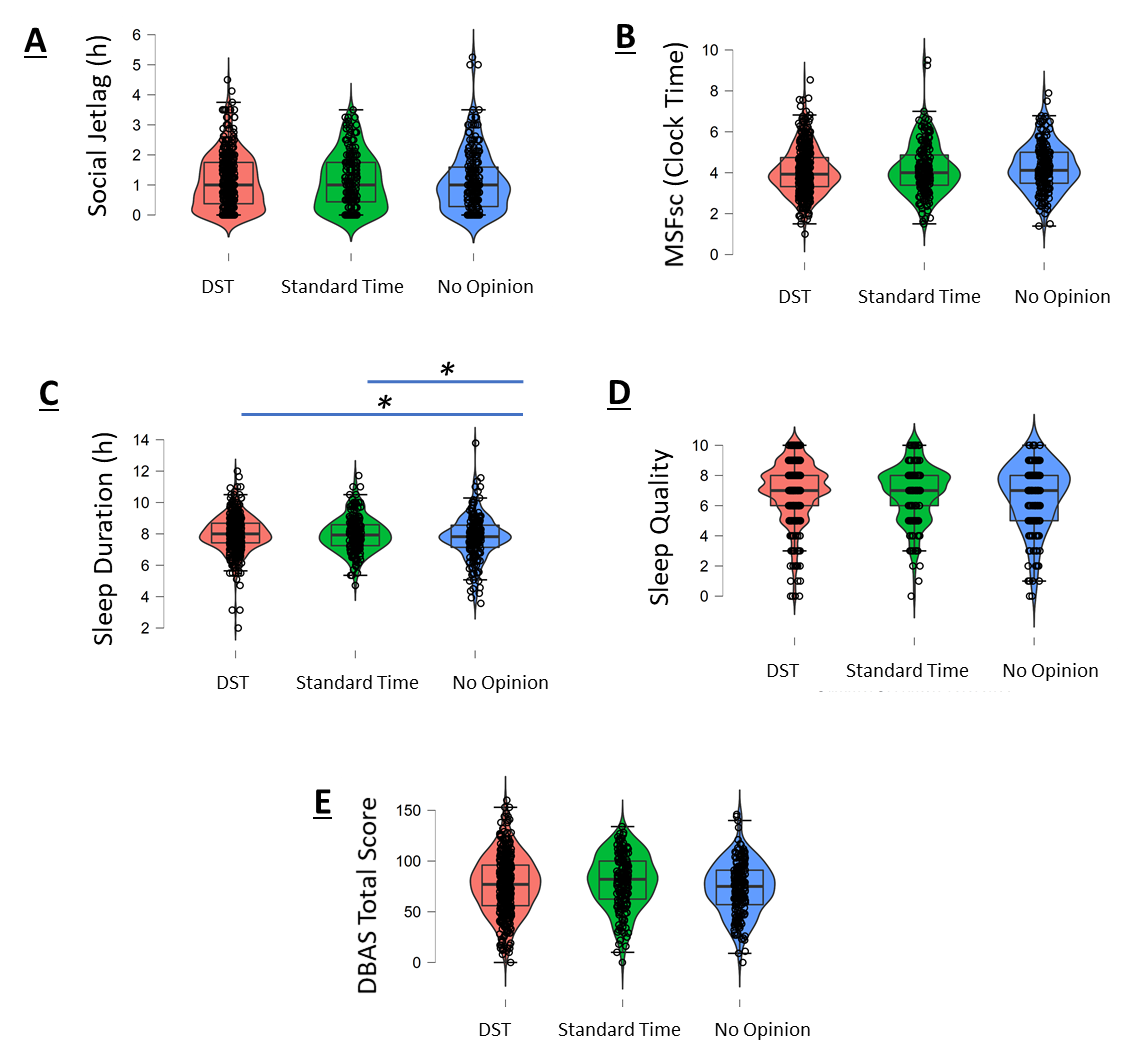


**Supplementary Figure11**: Box and Violin plots showing the distribution of (A) social jetlag, (B) MSFsc, (C) average weekly sleep duration, (D) subjective sleep quality and (E) DBAS scores in participants who favored abolition (n=452) according to whether they favored adoption of permanent DST, permanent standard time or with no expressed preference. P* indicates P<0.05.

**
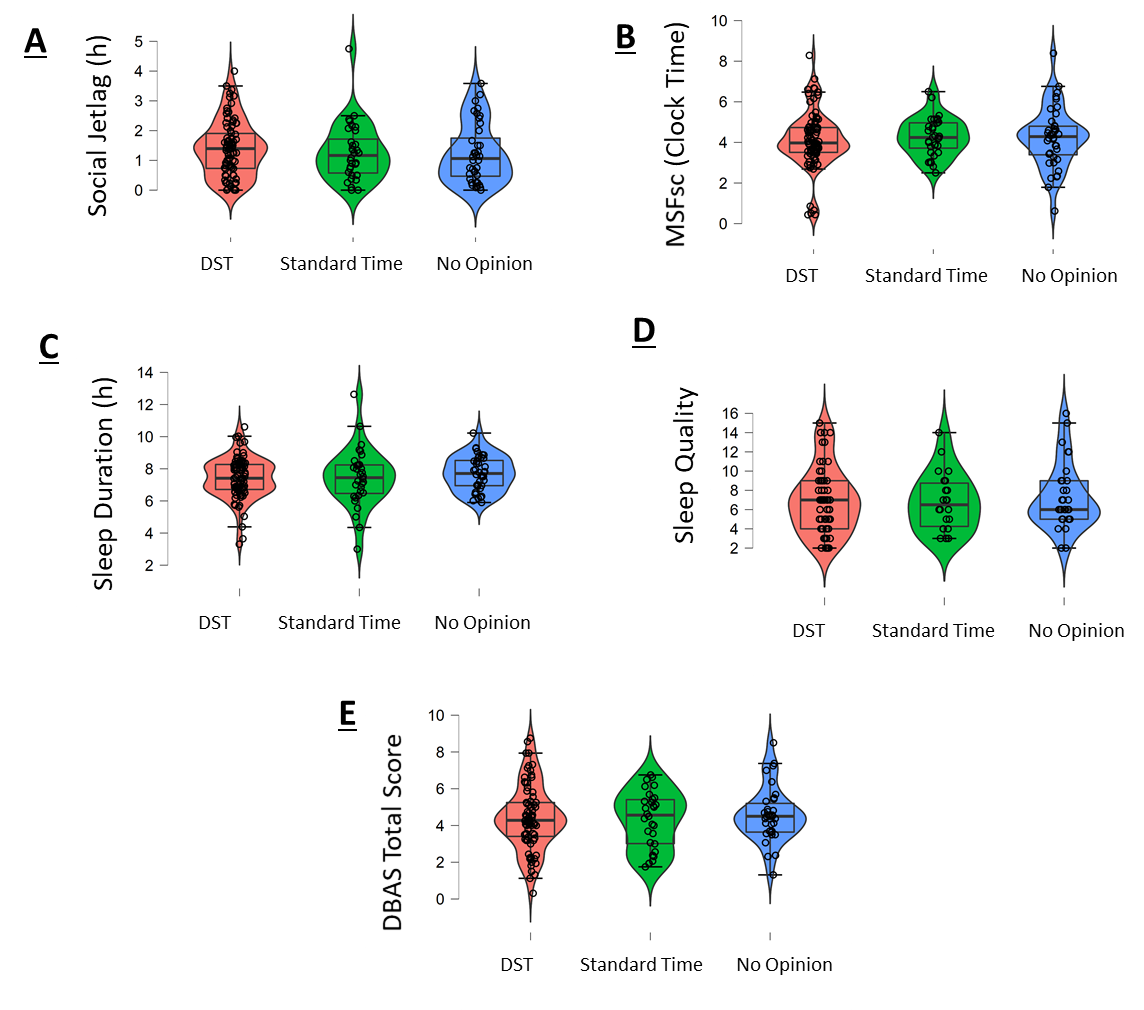
**

**Supplementary Figure 12:** From the Fall 2019 pilot study, box and violin plots showing the distribution of (A) social jetlag, (B) MSFsc, (C) average weekly sleep duration, (D) subjective sleep quality as measured by overall PSQI score and (E) DBAS scores in participants according to whether they favored adoption of permanent DST, permanent standard time or with no expressed preference if the clock changes were abolished (N=172).

**Survey assessing experiences of, and attitudes to, clock changes.**

The following questions refer to the advancing of the clock in spring (referred to as summertime) and reverting to standard time in fall (referred to as wintertime).

Q1. What is your overall experience with the switching from wintertime to summertime on the last Sunday of March? (The clocks go forward 1 hour)

- Very positive
- Positive
- No opinion
- Negative
- Very negative

Q2. What is your overall experience with switching from summertime back to wintertime on the last Sunday of October? (The clocks go back 1 hour)

- Very Positive
- Positive
- No opinion
- Negative
- Very Negative

Q3. Which of the following alternatives would you prefer?

- keep the current switching between summer and wintertime
- abolish the switching

Q4. Based on your response above, what is the most important reason for your choice

- Energy saving or lack of energy saving
- Human health
- Road safety
- Functioning of internal markets and cross border trade, transport, organization and communications
- Leisure activities in the evening
- Others

Briefly state what is the "others" reason:

_________________________________

Q5 Based on your response to if you would prefer to keep or abolish the switching, how important is it for you to see that happening?  
(0=not an important issue; 10=very important issue)

- 0 Not Important
- 1
- 2
- 3
- 4
- 5
- 6
- 7
- 8
- 9
- 10 Very Important

Q6. If the switching were to be abolished, what option would you prefer? To abolish the switching and stick with:

- Permanent summertime (clocks are advanced one hour from wintertime)
- Permanent wintertime/standard time
- no opinion/I don't know
